# A masked initiation region in retinoblastoma protein regulates its proteasomal degradation

**DOI:** 10.1038/s41467-020-16003-3

**Published:** 2020-04-24

**Authors:** Takuya Tomita, Jon M. Huibregtse, Andreas Matouschek

**Affiliations:** Department of Molecular Biosciences, The University of Texas at Austin, Austin, TX 78712 USA

**Keywords:** Proteasome, Proteasome

## Abstract

Retinoblastoma protein (Rb) is a tumor suppressor that binds and represses E2F transcription factors. In cervical cancer cells, human papilloma virus (HPV) protein E7 binds to Rb, releasing it from E2F to promote cell cycle progression, and inducing ubiquitination of Rb. E7-mediated proteasomal degradation of Rb requires action by another protease, calpain, which cleaves Rb after Lys 810. However, it is not clear why cleavage is required for Rb degradation. Here, we report that the proteasome cannot initiate degradation efficiently on full-length Rb. Calpain cleavage exposes a region that is recognized by the proteasome, leading to rapid proteolysis of Rb. These findings identify a mechanism for regulating protein stability by controlling initiation and provide a better understanding of the molecular mechanism underlying transformation by HPV.

## Abstract (web summary)

Human papilloma virus (HPV) E7 protein destabilizes the retinoblastoma protein (Rb) by inducing its ubiquitination in cervical cancer cells, however proteasomal degradation requires cleavage of Rb after Lys 810 and so far it has been unclear how Rb cleavage contributes to its degradation. Here, the authors combine cell based and in vitro assays and show that calpain cleavage exposes a region in Rb that is recognized by the proteasome, leading to rapid proteolysis of Rb, whereas the proteasome cannot initiate degradation efficiently on full-length Rb.

## Introduction

Most regulated intracellular protein degradation is conducted by the ubiquitin-proteasome system (UPS)^1^. Proteins are targeted to the proteasome degradation by the covalent attachment of several copies of the small protein ubiquitin through a cascade of three classes of enzymes: ubiquitin-activation enzyme (E1), ubiquitin-conjugating enzymes (E2), and ubiquitin ligases (E3)^2^. The resulting poly-ubiquitin chains are recognized by receptors on the proteasome^3^. Ubiquitination is tightly controlled to adjust the abundance of regulatory proteins and other UPS substrates. However, ubiquitination by itself is not sufficient for proteasomal degradation. Efficient degradation requires the presence of an unstructured region at which the proteasome can engage its substrate and initiate unfolding and degradation^4,5^. The effectiveness of an initiation region is determined by several properties including its location, length, and amino acid composition^5^. The initiation step can also contribute to substrate selection, and some proteins escape degradation despite binding to the proteasome because the proteasome fails to initiate degradation^6–8^.

Viruses have found mechanisms to subvert the UPS to transform host cells^9^. For example, human papilloma virus (HPV) deploys its E6 protein to hijack the cellular E3 ubiquitin ligase E6AP to destroy the tumor suppressor p53 and stimulate cell proliferation^10^. HPV also inactivates another tumor suppressor, retinoblastoma protein (Rb), through its E7 protein^11^. Rb binds and represses E2F transcription factors, which drive expression of S phase genes, thereby arresting cell cycle progression^12^. HPV E7 protein binds to Rb, which releases E2F transcription factors to promote cell cycle progression^13,14^. In addition, high-risk HPV E7 such as type 16 E7 (16E7) also decreases the half-life and steady-state level of Rb by inducing its proteasomal degradation. Treatment of cells expressing E7 with proteasome inhibitors increases Rb levels^15^, and the E7 is thought to induce Rb ubiquitination by cullin E3 ligases^16,17^. E7-mediated Rb degradation further requires the action of the protease calpain-1 (also called μ-calpain)^18^, which belongs to a group of calcium-activated cysteine proteases and plays a role in the regulation of multiple cellular processes^19^. E7 activates and recruits calpain-1 to Rb to induce cleavage after residue Lys 810, and the cleavage product Rb^1–810^ can be detected in vitro and in some HPV-positive cells including HeLa and Caski cells after treatment with proteasome inhibitor^18^. The Rb^1–810^ fragment does not bind E2F transcription factors and is unstable in cells. However, it is not known how this cleavage destabilizes Rb.

Here, we report that calpain cleavage exposes a region in Rb that is recognized by the proteasome and allows it to initiate degradation efficiently, thereby significantly reducing its stability. We found that full-length Rb was stabilized in cells, probably because the negatively charged amino acid sequence at its C-terminus prevents the proteasome from initiating degradation. The molecular mechanism of E7-mediated destabilization of cleaved Rb we present in this study thus introduces a regulatory mechanism for selective degradation of cellular proteins by the proteasome.

## Results

### Calpain and a cullin E3 ligase contribute to Rb degradation

**Fig. 1:**
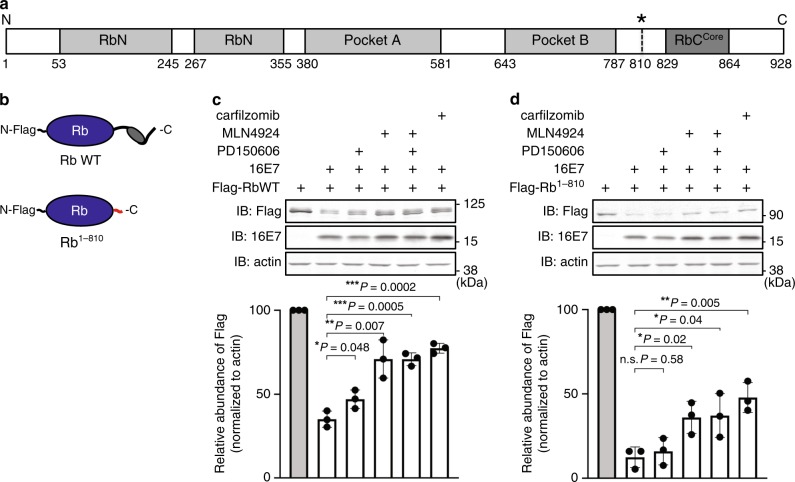
Contribution of calpain and a cullin E3 ligase to Rb degradation. **a** Schematic representation of Rb’s domain structure. Rb contains two structured domains (RbN and Pocket A/B, gray), which are flanked by disordered regions including RbC^Core^ (dark gray) in the C-terminal region. The asterisk denotes the calpain cleavage residue Lys 810. **b** Schematic view of N-terminally Flag-tagged full-length Rb protein (Rb WT) and the cleavage product (Rb^1–810^). The RbC^Core^ and cleavage-exposed tail are highlighted in dark gray and red, respectively. **c, d** Representative images of immunoblots of Rb mutants in the presence of 16E7 and inhibitors. HEK293T cells were transfected with Rb WT (**c**) and Rb^1–810^ (**d**), as well as 16E7 or empty vector as indicated, for 24 h. Cells were treated with vehicle (DMSO), 100 μM PD150606, 1 μM MLN4924, and/or 1 μM carfilzomib for the last 4 h, and the lysates were subjected to immunoblotting with the indicated antibodies. The graph plots the relative band intensities as means ± the standard deviations (SD) from three repeat experiments. ns not significant. *P < 0.05; **P < 0.01; ***P < 0.001 (two-tailed unpaired *t*-test). Source data are provided as a Source Data file.

Rb is a 928-amino-acid protein, which contains two folded domains, an N-terminal domain (RbN) and a pocket domain (Pockets A and B), both of which consist of two helical subdomains^12^ (Fig. 1a). The subdomains are flanked by intrinsically disordered regions as determined by the crystal structure, including an N-terminal stretch that precedes RbN, internal loops between the Pockets A and B subdomains, and most of the C-terminal domain (RbC) following Pocket B. RbC forms a helix-turn-strand structure in the RbC^Core^ region. HPV E7 binds to Pocket B through an LxCxE amino acid sequence motif^20^. The calpain cleavage site at Lys 810 is located in a disordered region flanked by Pocket B and RbC^Core^^18^.

E7 is thought to destabilize Rb by inducing its ubiquitination and cleavage by calpain and we first validated these factors in our culture cell system using inhibitors. We constructed expression vectors for N-terminally Flag-tagged wild-type Rb (Rb WT), and truncated mutant Rb (Rb^1–810^) mimicking its calpain cleaved form (Fig. 1b), as well as a second vector for expression of 16E7. HEK293T cells express adenovirus E1A protein and simian virus 40 large T antigen, both of which contain the LxCxE motif that can bind Rb but neither is thought to induce Rb degradation^21^. Despite the presence of these proteins, Rb protein levels were reduced upon co-expression of 16E7 (Fig. 1c). Treatment with the calpain inhibitor PD150606 restored Rb protein levels partially, indicating that calpain activity contributed to the 16E7-mediated destabilization of Rb, as reported^18^. Treatment with the NEDD8-activation inhibitor MLN4924, which inhibits cullin E3 ligases, also stabilized Rb, as expected^17^. Rb degradation was by the proteasome as treatment with the proteasome-specific inhibitor carfilzomib reversed its destabilization by 16E7. We were unable to detect the cleavage product (Rb^1–810^) in the presence of proteasome inhibitor, presumably because it was rapidly ubiquitinated.

Overexpression of 16E7 also destabilized the Rb fragment (Rb^1–810^), which was suppressed by E3 ubiquitin ligase inhibitor (MLN4924) and proteasome inhibitor (carfilzomib) (Fig. 1d). However, calpain inhibitor (PD150606) did not stabilize Rb fragment, presumably because it represented already cleaved Rb, illustrating the destabilizing effect of calpain cleavage. Collectively, these results suggest that both calpain cleavage at Lys 810 and, as expected, ubiquitination through cullin E3 ligases, participate in the destabilization of Rb by 16E7^16–18^.

### Rb degradation is initiated at a tail exposed by cleavage

The observation that Rb cleavage plays a part in its degradation motivated us to search for destabilizing factors other than ubiquitination. The proteasome requires disordered regions in its substrates to initiate degradation^4,5^, and we noticed that calpain cleavage exposes an unstructured region in Rb (see Fig. 1a). In fact, a region within the C-terminal region that covers the cleavage site has been reported to function as a degron^22^, yet how this element destabilizes full-length Rb remains elusive. We therefore hypothesized that the cleavage destabilizes Rb by allowing the proteasome to initiate degradation on Rb.

**Fig. 2:**
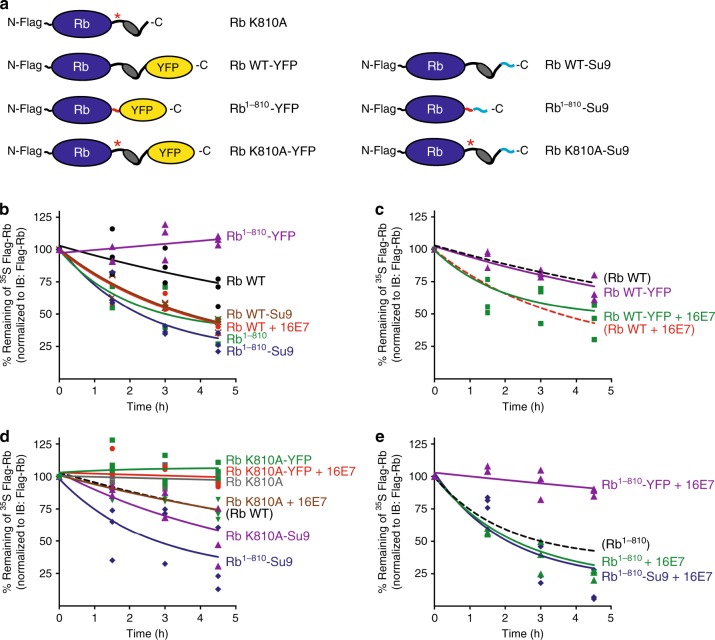
The degradation of Rb is regulated by its C-terminus. **a** Schematic view of Rb WT, Rb^1–810^, and the cleavage-resistant mutant of Rb in which Lys 810 was replaced with Ala (Rb K810A), with either YFP or an Su9 tail attached at their C-termini. The K810A mutation site, YFP, and Su9 tail are highlighted in red with an asterisk, in yellow, and in light blue, respectively. **b**–**e** Metabolic pulse chase of Rb in cells. HEK293T cells were transfected with Rb mutants (see Fig. 1b and **a** of the figure) and empty vector or 16E7 for 42 h. The cells were metabolically labeled with [^35^S] methionine/cysteine for the last 20 h, washed twice with PBS, and chased in label-free medium for the indicated times. Rb mutants were immunoprecipitated from the cell lysates and subjected to SDS-PAGE, followed by immunoblotting with Flag antibody and electronic autoradiography. The effects of modifications at the Rb C-terminus (**b**), of 16E7 expression (**c**, **e**), and of the K810A mutation (**d**) on the degradation rates of Rb were evaluated. Degradation of Rb WT, Rb WT + 16E7, and Rb^1–810^ were replotted in some panels with broken lines to help with comparisons. The graphs plot the relative band intensities over time as a percentage of the initial protein amount from three repeat experiments. Representative gel images are shown in Supplementary Fig. 1a. Source data are provided as a Source Data file.

To investigate the contribution of the initiation step to Rb degradation, we constructed a further set of Rb mutants including the K810A mutant of Rb in which Lys 810 was replaced with Ala (Rb K810A), which is resistant to calpain cleavage^18^, and measured their degradation in HEK293T cells through ^35^S pulse-chase experiments (Fig. 2a and Supplementary Fig. 1a). Overexpressed Rb (Rb WT) was slowly degraded by the proteasome and co-expression of 16E7 stimulated the degradation (Fig. 2b). Mimicking calpain cleavage by deleting the C-terminal 118 amino acids of Rb exposed a disordered tail at the new C-terminus and accelerated degradation substantially (Rb^1–810^; Fig. 2b). Blocking the exposed tail by fusing a bulky domain (yellow fluorescent protein, YFP) to its end inhibited degradation (Rb^1–810^-YFP; Fig. 2b). At the same time, attaching a disordered region that is recognized by the proteasome (Su9^23^) to full-length Rb (Rb WT-Su9) accelerated degradation, but attaching this tail to truncated Rb (Rb^1–810^-Su9) had only a small effect, if any (Fig. 2b).

Overexpression of 16E7 accelerated Rb degradation even when the C-terminus was blocked with YFP (Rb WT-YFP), presumably because the YFP is cleaved off by calpain together with the rest of the C-terminus of Rb (Fig. 2c). Inhibiting calpain cleavage of Rb by mutating Lys 810 to Ala (Rb K810A) attenuated E7-induced degradation as expected^18^, but attaching a disordered tail to the K810A mutant restored degradation (Rb K810A-Su9), presumably because the tail allowed the proteasome to initiate degradation even without cleavage (Fig. 2d). These observations indicate that the C-terminal end of Rb physically blocks degradation by preventing the proteasome from engaging the protein. Creating a disordered region at the C-terminus or providing it artificially overcomes this inhibition.

Rb was constitutively degraded slowly, and the mutation K810A stabilized Rb completely (Fig. 2d), consistent with the observation that calpains cleave Rb to some extent in the absence of E7^24^. Overexpression of 16E7 stimulated degradation of the cleavage-resistant Rb (Rb K810A), presumably by stimulating its ubiquitination^16,17^, which can compensate for and overcome inefficient initiation^23^. Blocking initiation by fusing a YFP domain to the C-terminus stabilized the protein again (Rb K810A-YFP). Apparently, the C-terminal domain of full-length Rb allows some residual initiation of proteasomal degradation, which becomes noticeable when ubiquitination is stimulated. 16E7 also enhanced degradation of Rb with a disordered tail (Rb-Su9) (Supplementary Fig. 1b) and of cleavage-resistant Rb with a disordered tail (Rb K810A-Su9) (Fig. 2d), presumably again because 16E7 stimulated their ubiquitination^16,17^. Similarly, 16E7 overexpression enhanced degradation of truncated Rb (Rb^1–810^ and Rb^1–810^-Su9), unless proteasome initiation was blocked again by fusion of YFP domain to its C-terminus (Rb^1–810^-YFP + 16E7; Fig. 2e).

In conclusion, HPV E7 stimulated Rb degradation through two mechanisms: by enhancing Rb ubiquitination and by exposing a proteasome initiation site through calpain cleavage. The proteasome does not degrade full-length Rb efficiently because it is unable to engage the protein at its C-terminus. Rb cleavage by calpain reveals a new C-terminus that is more efficiently recognized by the proteasome leading to Rb’s degradation.

### The initiation region in Rb is masked by the C-terminal end

**Fig. 3:**
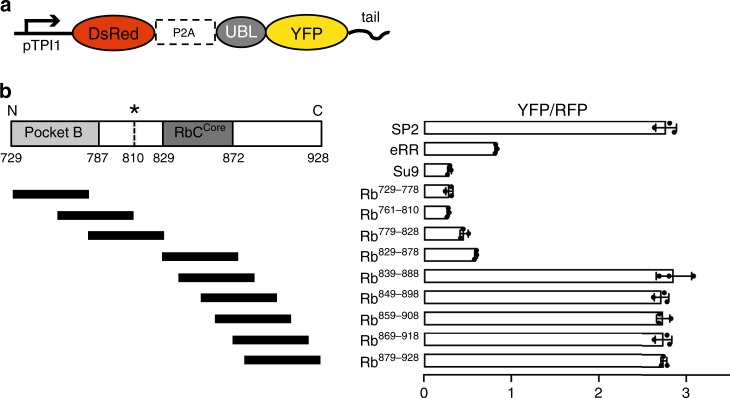
Proteasome initiation at different Rb sequences. **a** Schematic representation of the fluorescence-based degradation assay in *S. cerevisiae*. The substrate proteins consisted of an N-terminal UBL domain derived from *S. cerevisiae* Rad23, followed by a YFP domain and a tail. Genes encoding DsRed (RFP) followed by the ribosome skipping sequence P2A and the coding sequence for YFP were expressed from the same promoter (pTPI1) on the same plasmid to produce the RFP reference and YFP substrates at a constant ratio. **b** Cell fluorescence profiles of *S. cerevisiae* cultures expressing proteasome substrates with different initiation regions monitored by flow cytometry. UBL-YFP substrates with control initiation regions (SP2, eRR, and Su9) or 50 amino acid sequences derived from Rb as indicated. The graph shows standardized median cellular YFP fluorescence (median YFP/RFP values) ± SD from triplicate experiments. Data are representative of three independent experiments. Source data are provided as a Source Data file.

The results above suggest that degradation of Rb is modulated at the initiation step of proteasomal degradation. Therefore, we next screened the sequences within the C-terminus of Rb for their ability to function as initiation regions directly using model substrates. The proteasome’s sequence preferences are conserved between yeast and human cells^23^, which allowed us to investigate initiation in the versatile yeast system. We constructed a set of plasmids that express a proteasome substrate derived from YFP and a red fluorescent protein (RFP) as a reference at a constant ratio by driving transcription of the coding sequences of the YFP and RFP constructs separated by P2A ribosome skipping site from a single constitutive promoter (pTPI1) (Fig. 3a). The YFP construct was targeted to the proteasome by the ubiquitin-like domain (UBL) of yeast Rad23 fused to its N-terminus. UBL-YFP is only degraded when it also contains a sequence that allows the proteasome to initiate degradation fused to its C-terminus^23^. RFP served as an internal reference for normalization. The ratio of YFP over RFP fluorescence of individual cells therefore represents the effectiveness of degradation of the YFP substrates. YFP degradation, in turn, is determined by how well their C-terminal tails allow the proteasome to initiate degradation^23^.

We fused UBL-YFP with several 50 amino acid long regions derived from Rb C-terminal sequences and compared their ability to drive proteasomal degradation with three reference sequences (Su9, eRR, and SP2, which allow effective, medium, and ineffective initiation, respectively^23^) (Supplementary Table 1 and Fig. 3b). The C-terminal end of Rb (Rb^879–928^) allowed proteasomal degradation as poorly as the negative control (SP2), suggesting that the proteasome cannot engage the Rb C-terminus effectively. In contrast, the Rb region exposed by calpain cleavage (Rb^761–810^) allowed degradation as well as the positive control sequence (Su9). The regions surrounding the calpain cleavage site worked similarly well (Rb^729–778^, Rb^761–810^, Rb^779–828^, and Rb^829–878^), whereas regions closer to the C-terminal end of Rb (Rb^839–888^, Rb^849–898^, Rb^859–908^, Rb^869–918^, and Rb^879–928^) did not support degradation.

We further asked whether the Rb region exposed by calpain cleavage (Rb^761–810^) contained ubiquitination signals that would affect degradation of the model proteins. We removed the proteasome-binding tag by replacing the UBL domain with an unrelated protein, barstar from *B. amyloliquefaciens*, so that the new protein (Barstar-YFP-Rb^761–810^) contained no known proteasome-binding sequences. We also constructed a version with the native Rb tail (Barstar-YFP-Rb^879–928^). We then expressed these proteins in a yeast strain (*uba1-204*) carrying a temperature-sensitive mutant of the yeast E1 gene, which allowed us to reduce protein ubiquitination drastically by shifting the cells to the restrictive temperature^25^ (Supplementary Fig. 2a). In this system, a control substrate with a classical ubiquitination signal (degron) (R-KK-YFP-Su9^26^) was stabilized approximately fourfold at the restrictive temperature, while a mutant with a strongly attenuated ubiquitination signal (V-KK-YFP-Su9^26^) accumulated to the same extent at the permissive and restrictive temperatures (Supplementary Fig. 2b). YFP substrate with the native Rb tail (Barstar-YFP-Rb^879–928^) behaved like the ubiquitination-defective control (V-KK-YFP-Su9) and accumulated at both temperatures. Surprisingly though, the substrate with the C-terminal end of Rb created by calpain cleavage (Barstar-YFP-Rb^761–810^) was degraded at both the permissive restrictive temperatures suggesting that degradation did not depend on ubiquitination (Supplementary Fig. 2b). Degradation was by the proteasome, as YFP fluorescence increased in the presence of the proteasome inhibitor bortezomib (Supplementary Fig. 2c). These results indicate that the amino acid sequence exposed by calpain cleavage is recognized so well by the proteasome in yeast and that it can target proteins to degradation independent of ubiquitination, similar to an established ubiquitin-independent degron derived from ornithine decarboxylase (ODC)^27,28^.

### The unmasked Rb tail serves as an efficient initiation site

**Fig. 4:**
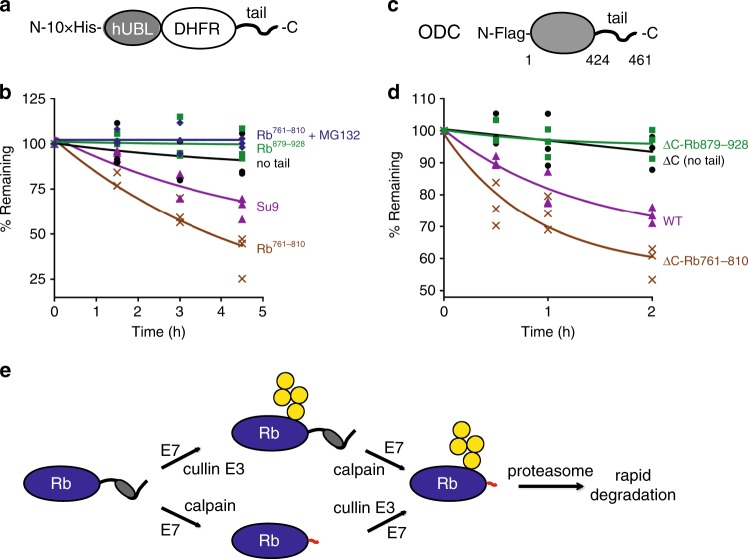
The unmasked tail of Rb serves as an effective initiation region in vitro. **a** Sketch of proteasome substrates consisting of the UBL domain of human HR23B with 10×His-tag at the N-terminus, followed by an *E. coli* DHFR domain and a tail (10×His-hUBL-DHFR-tail). **b** Degradation of 10×His-hUBL-DHFR-tail containing different C-terminal tails as indicated by purified mammalian 26S proteasomes. 100 μM MG132 was added to the reactions where indicated. The graph plots the relative amount of protein estimated by electronic autoradiography in SDS-PAGE gel bands (representative images are shown in Supplementary Fig. 3b) over time as a percentage of the initial protein amount from three repeat experiments. **c** Schematic representation of Flag-tagged human ODC protein. ODC contains an unstructured region at its C-terminal end. **d** Degradation of ODC and the indicated variants by purified mammalian 26S proteasomes as in **b**: Flag-tagged ODC (WT), Flag-tagged ODC in which the C-terminal tail (amino acids 425–461) is deleted (∆C (no tail)), Flag-tagged ODC in which amino acids 425–461 are replaced with amino acids 879-928 of Rb (∆C-Rb^879–928^), and Flag-tagged ODC in which amino acids 425–461 are replaced with amino acids 761–810 of Rb (∆C-Rb^761–810^). Representative gel images are shown in Supplementary Fig. 3d. The graph plots the relative band intensities from three repeat experiments. **e** Proposed model of E7-mediated destabilization of Rb. E7 induces Rb cleavage and also enhances its ubiquitination, both of which facilitate degradation of Rb by the proteasome. Exposure of the intrinsic disordered region of Rb by calpain cleavage markedly accelerates proteasomal degradation of Rb because the exposed region serves as an effective initiation region for proteasomal degradation. Source data are provided as a Source Data file.

To investigate the interaction of Rb sequences with the proteasome unambiguously, we analyzed the degradation of model substrates by mammalian proteasome purified from mouse embryonic fibroblasts^29^. These substrates consisted of central *E. coli* dihydrofolate reductase (DHFR) domain, which was targeted to the proteasome by the UBL domain of HR23B (the human homolog of the yeast Rad23, hUBL) with an N-terminal decahistidine tag (10×His-tag) attached to the N-terminus of the UBL domain. We then fused potential initiation regions to the C-terminus of DHFR and performed degradation assays after expressing the constructs in vitro, affinity purifying them through their His-tag and presenting them to purified mammalian proteasomes (Fig. 4a, Supplementary Fig. 3a, b).

Attaching the effective initiation region Su9 to the hUBL-DHFR led to efficient degradation, while hUBL-DHFR without an initiation region (no tail) was degraded poorly (Fig. 4b). The cleavage-exposed region of Rb also supported rapid degradation (hUBL-DHFR-Rb^761–810^), whereas the native C-terminus of Rb did not allow degradation (hUBL-DHFR-Rb^879–928^). Degradation of hUBL-DHFR- Rb^761–810^ was dependent on the proteasome as treatment with the proteasome inhibitor MG132 stabilized the protein.

We also evaluated the effectiveness of Rb tails in initiating degradation in the context of a physiological proteasome substrate for which the importance of C-terminal sequences is firmly established. ODC controls polyamine biosynthesis and its degradation is induced in response to elevation of cellular polyamine levels^30^. ODC is degraded by the proteasome without ubiquitination but requires the cofactor antizyme^31^. Degradation also depends strictly on the presence of a 37 amino acid long disordered region at the C-terminus of ODC^27^ (Fig. 4c).

We generated wild-type ODC (WT) and three derivatives in which its C-terminal tail was either removed (∆C) or replaced with the native C-terminus of Rb (∆C-Rb^879–928^) or the Rb C-terminus generated by calpain cleavage (∆C-Rb^761–810^). We again expressed these proteins in vitro, purified them using the N-terminal Flag-tag, and followed their degradation by purified mouse proteasome in the presence of purified antizyme (Supplementary Fig. 3c). Consistent with previous reports, the WT ODC was degraded by the proteasome in the presence of antizyme, while ODC without its tail escaped degradation (Fig. 4d and Supplementary Fig. 3d). The ODC mutant whose tail was replaced with the cleavage-exposed Rb tail (∆C-Rb^761–810^) was efficiently degraded, but the mutant with the native Rb C-terminus (∆C-Rb^879–928^) was stable. These results demonstrate that the cleavage-exposed tail of Rb serves as an effective initiation region for proteasomal degradation and that exposure of this tail initiates proteasomal degradation of Rb (Fig. 4e).

## Discussion

We identified Rb as an example of a physiological protein the stability of which is regulated by controlling access to the proteasomal initiation region. So far, the regulation of proteasomal degradation has been explained by control of the extent of ubiquitination of substrates. For instance, the E3 ligase anaphase-promoting complex/cyclosome targets multiple cell cycle regulators for ubiquitination, yet the substrates are degraded in a specific order^32,33^. This so-called substrate ordering is accounted for by the differences in the rate with which the poly-ubiquitin chain is synthesized on each substrate. This view is expanded by the regulation of Rb degradation, which drastically changes its stability when an unstructured region is exposed to allow the proteasome to engage the protein. In this way, the quality of the initiation region of a substrate that is accessible to the proteasome determines the protein’s stability in cells.

Protein association with the proteasome is regulated by ubiquitination but proteasome-binding does not always lead to degradation. For example, the proteasome-interacting proteins Ubp6^8^ and Rad23^6^ in yeast escape degradation because they do not contain disordered regions that would be long enough to allow the proteasome to engage them for degradation. The proteasome also has strong preferences for the amino acid composition of the initiation region. Systematic studies with model proteins have shown that negative charge and biased amino acid composition in initiation regions correlate with poor degradation, whereas hydrophobicity tends to promote degradation^7,23^. For example, in yeast Cdc34 is not degraded even when ubiquitinated as the proteasome fails to engage the substrate at the only disordered region in the protein, apparently because its composition is strongly biased and the sequence is highly negatively charged^7^. We speculate that these rules may apply to Rb, considering that 4 of the last 11 amino acids of Rb’s C-terminal end (Rb^918–928^; highlighted in Supplementary Fig. 4) are acidic (two Asp and two Glu) and that it has few hydrophobic amino acids, which probably contribute to the tails’ stabilizing effect (Supplementary Table 1). Similarly, the stabilizing region Rb^879–888^ (highlighted in Supplementary Fig. 4, also see Fig. 3b) contains two Asp and two Glu out of ten amino acids, supporting stabilizing effect of acidic residues. The C-terminal tail exposed by calpain cleavage in contrast lacks negative charge and contains many hydrophobic amino acids (Supplementary Table 1), in alignment with the properties of effective initiation regions^23^.

Rb contains other disordered regions and the proteasome is able to initiate degradation to a small extent at these as we observed a slight destabilization of the uncleavable Rb mutant K810A upon overexpression of 16E7 (Fig. 2d). Nevertheless, initiation at these regions is quite inefficient, as reflected by the high stability of Rb in which the initiation at the site created by cleavage has been blocked (Rb^1–810^-YFP), even upon overexpression of 16E7 (Fig. 2b, d). In fact, the sequence of the 52 amino acid long disordered N-terminal tail of Rb is strongly biased and highly negatively charged (15 Pro, 9 Ala, and 7 Glu residues out of 52 amino acids), and its net charge at physiological pH is −7.4, which is substantially lower than that of Rb’s C-terminal tail (Rb^879–928^) (Supplementary Tables 1 and 2). The internal disordered loops in Rb are presumably too short (61 amino acids at most, see Fig. 1a) for efficient internal initiation, analogous to the situation found in Rad23^6^.

The native C-terminal end of Rb is one of the worst inefficient initiation regions observed, and the cleavage-exposed tail of Rb is the best initiation region that we have discovered so far, as effective as the C-terminal tail of ODC, which serves as a ubiquitin-independent degron, at least in some contexts. In contrast to many other cell cycle regulators, Rb activity is not regulated by degradation but by phosphorylation of Rb or competitive binding of HPV E7 is sufficient to release E2F transcription factors^12,20^. Rb is a classical tumor suppressor; it is inactivated in retinoblastoma and in many sporadic tumors, and it is targeted by small DNA tumor viruses^12^. Thus, it is clearly important for cell division control that the protein is maintained in the cell, which may explain why it consists of sequences that protect it against proteasomal degradation. Ubiquitination is widely used as a regulatory posttranslational modification in pathways where it does not signal degradation. Rb ubiquitination is detected in several high-throughput studies, and some sites are found consistently^34^, which suggests that Rb may be regulated in this manner, too. Even modification by single ubiquitin moieties can target protein for degradations^3^, reinforcing the need to protect essential proteins from accidental destruction.

Phosphorylation increases the negative charge of a sequence, which decreases the efficiency of initiation in this region and thus phosphorylation of the C-terminal tail presumably further stabilizes the protein. Therefore, cleaving off the C-terminal region may also contribute to reducing negative charges for efficient proteasomal degradation. We actually observed multiple bands in Rb WT but not in Rb^1–810^, which may have derived from phosphorylation as they diminished upon treatment with the alkaline phosphatase CIP (Supplementary Fig. 5, also see Fig. 1c, d and Supplementary Fig. 1a). When not phosphorylated, the C-terminus of Rb serves as a landing site for binding proteins including E2Fs and this interaction would again stabilize the protein against degradation similar to the observation that attachment of a folded protein domain stabilized Rb mutants (Fig. 2). Consequently, cleavage of the native Rb C-terminal tail has positive effects on destabilization of Rb in multiple ways, and HPV E7 proteins may efficiently induce transformation of host cells by destroying Rb, which is irreversible unlike phosphorylation.

Viruses evolve effective ways to subvert cellular mechanism for their benefits and there are many examples where viruses induce the rapid degradation of cellular proteins that impede their proliferation. The prototypical example of this mechanism is the destruction of the tumor suppressor p53 by the HPV E6 protein, which hijacks the cellular E3 ubiquitin ligase E6AP to induce p53 ubiquitination^10^. The activity of p53 is regulated by ubiquitination and degradation as part of its normal cellular physiology, making it susceptible to this mechanism. To destabilize Rb, besides ubiquitination, the virus has found another way to make a protein susceptible to degradation by unmasking an initiation region in cooperation with calpain. Our study revealed that protein degradation associated with HPV infection is also regulated by exposure of an effective initiation region. This mechanism may regulate the stabilities of other cellular proteins in cells.

## Methods

### Substrate proteins

Protein substrates were derived from human Rb, ODC, and *E. coli* DHFR, and fluorescence proteins used in this study were RFP (DsRed-Express2) and YFP (Venus for mammalian expression and sYFP2 for yeast expression). Venus was attached to the C-terminal end of Rb by the linker sequence LESGSGKPGS. N-terminal UBL domains from *S. cerevisiae* Rad23 or human HR23B, N-end rule degrons or *B. amyloliquefaciens* barstar were connected to sYFP2 or DHFR by the linker sequence VDGGSGGGS. C-terminal tails were attached through a 2-amino acid linker (Pro-Arg). The amino acid sequences of the tails are provided in Supplementary Table 1. The coding sequences were cloned into pcDNA3 or pIRES (Clontech) for culture cell transfection, into CEN plasmid YCplac33 for expression in yeast, or into pE3a (Novagen) for expression in vitro. The sequences of the primers used are given in Supplementary Table 3.

### Cell culture and transfection

HEK293T cells (ATCC #CRL-3216) were cultured in Dulbecco modified Eagle medium supplemented with 10% fetal bovine serum, 100 U/ml penicillin, 100 μg/ml streptomycin, and 2 mM L-glutamine at 37 °C with 5% CO_2_. The cells were transfected with the various expression vectors using an established calcium phosphate method^35^. To measure the half-life of Flag-tagged Rb mutants, transfected cells were metabolically labeled with [^35^S] methionine/cysteine for 20 h, washed twice with phosphate-buffered saline (PBS), and chased for the indicated times in regular medium. Cells were harvested, washed with PBS, and lysed with buffer A (20 mM Tris-HCl [pH 7.4], 0.2% [v/v] NP-40, 150 mM NaCl, and 1 mM DTT) containing protease inhibitor cocktail set V (Millipore #539137). The extract was clarified by centrifugation at 20,000 × *g* for 10 min at 4 °C. PD150606 (Sigma #D5946), MLN4924 (Sigma #505477), carfilzomib (UBPBio #F1300), bortezomib (LC Laboratories #B-1408), MG132 (Enzo #BML-PI102), CIP (New England Biolabs, #M0290), anti-Flag (1:1,000; Sigma #F1804), anti-actin (1:1,000; Sigma #A2066), anti-16E7 (1:1,000; Santa Cruz #sc-51951), and InstantBlue (Expedeon #ISB1L) were purchased from the indicated manufactures. Quantification of bands was performed using ImageJ (National Institutes of Health, version 1.51).

### Protein expression and purification

Mammalian proteasomes were purified from *Psmd14*^*Flag/Flag*^ mouse embryonic fibroblasts (Rpn11-Flag MEFs) by anti-Flag immunoaffinity chromatography essentially as described previously^29,36^. Rpn11-Flag MEFs were lysed with ice-cold buffer B (20 mM Tris-HCl [pH 7.4], 0.2% [v/v] NP-40, 150 mM NaCl, 1 mM dithiothreitol [DTT], 2 mM ATP, and 5 mM MgCl_2_), clarified by centrifugation (20,000 × *g* for 10 min at 4 °C), immunoprecipitated using anti-Flag M2 agarose affinity beads (Sigma #A2220). Proteasome was eluted with 100 μg/ml Flag peptide (Sigma #F3290) in buffer B. For in vitro degradation experiments, radioactive substrates were expressed from a T7 promoter using the TnT coupled in vitro transcription–translation reaction system (Promega) containing [^35^S] methionine/cysteine following the manufacturer’s protocol. After synthesis, substrates were affinity-purified using anti-Flag M2 agarose (for Flag-ODC) or TALON metal affinity resin (Clontech #635502; for 10×His-hUBL-DHFR) in NPI-10 buffer (50 mM NaH_2_PO_4_, 300 mM NaCl, and 10 mM imidazole [pH 8.0]) containing 0.05% (v/v) tween 20, 0.1 mg/ml bovine serum albumin (BSA) and 10 mM β-mercaptoethanol (BME), and eluted with NPI-250 buffer (50 mM NaH_2_PO_4_, 300 mM NaCl, and 250 mM imidazole [pH 8.0]) containing 0.1 mg/ml BSA, and 10 mM BME. The cDNA encoding 6×His-antizyme 1 (human AZ1) was cloned into pETDuet (Novagen) and expressed in Rosetta cells for purification using TALON resin as described above, but BSA was not added to NPI-10 and NPI-250 buffers. Purified proteins were supplemented with 10% glycerol for storage.

### Yeast transformation and flow cytometry

The CEN plasmids were transformed into either *S. cerevisiae* strain BY4741 (*MAT***a** *his3Δ1 leu2Δ0 met15Δ0 ura3Δ0*) or *uba1-204* with a *URA3* selection marker using Frozen-EZ yeast transformation II kit (Zymo Research #T2001). Proteasome inhibition experiments were performed using a BY4741 strain carrying a deletion of the efflux pump Pdr5 (*pdr5∆*). Yeast cells were cultured at 30 °C to early log phase before harvesting for analysis. Fluorescence intensities of RFP and YFP channels were measured by an LSR Fortessa (BD Biosciences) flow cytometer and analyzed by FlowJo (FlowJo, version 10.2) to calculate the median YFP over RFP fluorescence ratios of 10,000 cells in each population. Gating strategy is provided in Supplementary Fig. 6.

### Yeast ubiquitination assay

To ascertain the effect of reduced ubiquitination, we expressed control YFP substrates fused to an N-end rule degron consisting of a ubiquitin domain followed by a destabilizing (Arg) or stabilizing (Val) residue and a linker that contains two Lys residues (denoted as R-KK-YFP-Su9 and V-KK-YFP-Su9) (see Supplementary Fig. 2a)^26^. Following cleavage of the ubiquitin domain by endogenous deubiquitinases in the cells, an Arg residue that is exposed at the first amino acid of the fusion protein leads to substantial ubiquitination of the degron, whereas a Val leads to little ubiquitination. To inhibit ubiquitination in the *uba1-204* strain, cultures were incubated at 37 °C for 1 h and immediately subjected to measurement.

### In vitro degradation assays

Degradation assay with radiolabeled substrates was conducted at 37 °C by adding radiolabeled substrates to 7.5 nM of purified proteasomes in a reaction buffer C (50 mM Tris-HCl [7.4], 5 mM MgCl_2_, 2.5% [v/v] glycerol, 1 mM DTT, 1 mM ATP, 10 mM creatine phosphate, and 0.1 mg/ml creatine kinase). SDS-PAGE sample buffer was added to stop the reaction at each time point and samples were analyzed by SDS-PAGE. Protein amounts were determined by electronic autoradiography (Typhoon FLA 9500, GE Healthcare). The rate constant of the decay curve was determined by nonlinear fitting to a single exponential decay to a constant offset using the software package Prism8 (Graphpad, version 8.4.1).

### Bioinformatics

The physicochemical properties of tail sequences were calculated using the SnapGene software (GSL Biotech, version 4.2) for physiological charge and the ProtParam in ExPASy for theoretical pI and hydrophobicity.

### Reporting summary

Further information on research design is available in the Nature Research Reporting Summary linked to this article.

## Supplementary information

Supplementary Information

Reporting Summary

## Data Availability

The source data underlying Figs. 1c, d, 2b–e, 3b, 4b, d and Supplementary Figs. 1a, b, 2b, c, 3b, d, 5 are provided as a Source Data file. Other data that support the findings of this study are available from the corresponding author upon reasonable request.

## Source data

Source Data

## References

[1] Finley D, Prado MA (2020). The proteasome and its network: engineering for adaptability. Cold Spring Harb. Perspect. Biol.

[2] Deshaies RJ, Joazeiro CAP (2009). RING domain E3 ubiquitin ligases. Annu. Rev. Biochem..

[3] Saeki Y (2017). Ubiquitin recognition by the proteasome. J. Biochem..

[4] Prakash S, Tian L, Ratliff KS, Lehotzky RE, Matouschek A (2004). An unstructured initiation site is required for efficient proteasome-mediated degradation. Nat. Struct. Mol. Biol..

[5] Tomita T, Matouschek A (2019). Substrate selection by the proteasome through initiation regions. Protein Sci..

[6] Fishbain S, Prakash S, Herrig A, Elsasser S, Matouschek A (2011). Rad23 escapes degradation because it lacks a proteasome initiation region. Nat. Commun..

[7] Fishbain S (2015). Sequence composition of disordered regions fine-tunes protein half-life. Nat. Struct. Mol. Biol..

[8] Yu H, Kago G, Yellman CM, Matouschek A (2016). Ubiquitin-like domains can target to the proteasome but proteolysis requires a disordered region. EMBO J..

[9] Luo H (2016). Interplay between the virus and the ubiquitin–proteasome system: molecular mechanism of viral pathogenesis. Curr. Opin. Virol..

[10] Scheffner M, Huibregtse JM, Vierstra RD, Howley PM (1993). The HPV-16 E6 and E6-AP complex functions as a ubiquitin-protein ligase in the ubiquitination of p53. Cell.

[11] Burkhart DL, Sage J (2008). Cellular mechanisms of tumour suppression by the retinoblastoma gene. Nat. Rev. Cancer.

[12] Dick FA, Rubin SM (2013). Molecular mechanisms underlying RB protein function. Nat. Rev. Mol. Cell Biol..

[13] Münger K, Howley PM (2002). Human papillomavirus immortalization and transformation functions. Virus Res..

[14] Moody CA, Laimins LA (2010). Human papillomavirus oncoproteins: pathways to transformation. Nat. Rev. Cancer.

[15] Boyer SN, Wazer DE, Band V (1996). E7 protein of human papilloma virus-16 induces degradation of retinoblastoma protein through the ubiquitin-proteasome pathway. Cancer Res..

[16] Huh K (2007). Human papillomavirus type 16 E7 oncoprotein associates with the cullin 2 ubiquitin ligase complex, which contributes to degradation of the retinoblastoma tumor suppressor. J. Virol..

[17] White EA (2012). Systematic identification of interactions between host cell proteins and E7 oncoproteins from diverse human papillomaviruses. Proc. Natl Acad. Sci..

[18] Darnell GA (2007). Human papillomavirus E7 requires the protease calpain to degrade the retinoblastoma protein. J. Biol. Chem..

[19] Ono Y, Sorimachi H (2012). Calpains—an elaborate proteolytic system. Biochim. Biophys. Acta..

[20] Lee JO, Russo AA, Pavletich NP (1998). Structure of the retinoblastoma tumour-suppressor pocket domain bound to a peptide from HPV E7. Nature.

[21] Helt A-M, Galloway DA (2003). Mechanisms by which DNA tumor virus oncoproteins target the Rb family of pocket proteins. Carcinogenesis.

[22] Sengupta S (2015). The evolutionarily conserved C-terminal domains in the mammalian retinoblastoma tumor suppressor family serve as dual regulators of protein stability and transcriptional potency. J. Biol. Chem..

[23] Yu H (2016). Conserved sequence preferences contribute to substrate recognition by the proteasome. J. Biol. Chem..

[24] Tonnetti L (2008). SerpinB2 protection of retinoblastoma protein from calpain enhances tumor cell survival. Cancer Res..

[25] Ghaboosi N, Deshaies RJ (2007). A conditional yeast E1 mutant blocks the ubiquitin-proteasome pathway and reveals a role for ubiquitin conjugates in targeting Rad23 to the proteasome. Mol. Biol. Cell.

[26] Bachmair A, Varshavsky A (1989). The degradation signal in a short-lived protein. Cell.

[27] Takeuchi J, Chen H, Coffino P (2007). Proteasome substrate degradation requires association plus extended peptide. EMBO J..

[28] Erales J, Coffino P (2014). Ubiquitin-independent proteasomal degradation. Biochim. Biophys. Acta.

[29] Tomita T (2019). Specific modification of aged proteasomes revealed by tag-exchangeable knock-in mice. Mol. Cell. Biol..

[30] Coffino P (2001). Regulation of cellular polyamines by antizyme. Nat. Rev. Mol. Cell Biol..

[31] Murakami Y (1992). Ornithine decarboxylase is degraded by the 26S proteasome without ubiquitination. Nature.

[32] Rape M, Reddy SK, Kirschner MW (2006). The processivity of multiubiquitination by the APC determines the order of substrate degradation. Cell.

[33] Lu D (2014). Multiple mechanisms determine the order of APC/C substrate degradation in mitosis. J. Cell Biol..

[34] Hornbeck PV (2015). PhosphoSitePlus, 2014: mutations, PTMs and recalibrations. Nucleic Acids Res..

[35] Chen C, Okayama H (1987). High-efficiency transformation of mammalian cells by plasmid DNA. Mol. Cell. Biol..

[36] Martinez-Fonts K (2020). The proteasome 19S cap and its ubiquitin receptors provide a versatile recognition platform for substrates. Nat. Commun..

