# Supplementary material for: A masked initiation region in retinoblastoma protein regulates its proteasomal degradation
Source: Nat Commun. 2020 Apr 24;11:2019. doi: 10.1038/s41467-020-16003-3 (PMC7181824; doi:10.1038/s41467-020-16003-3)
Supplement: Supplementary file 1 — Supplementary Information [file 41467_2020_16003_MOESM1_ESM.pdf]

Supplementary Information

A masked initiation region in retinoblastoma protein regulates its proteasomal degradation

T. Tomita et al.

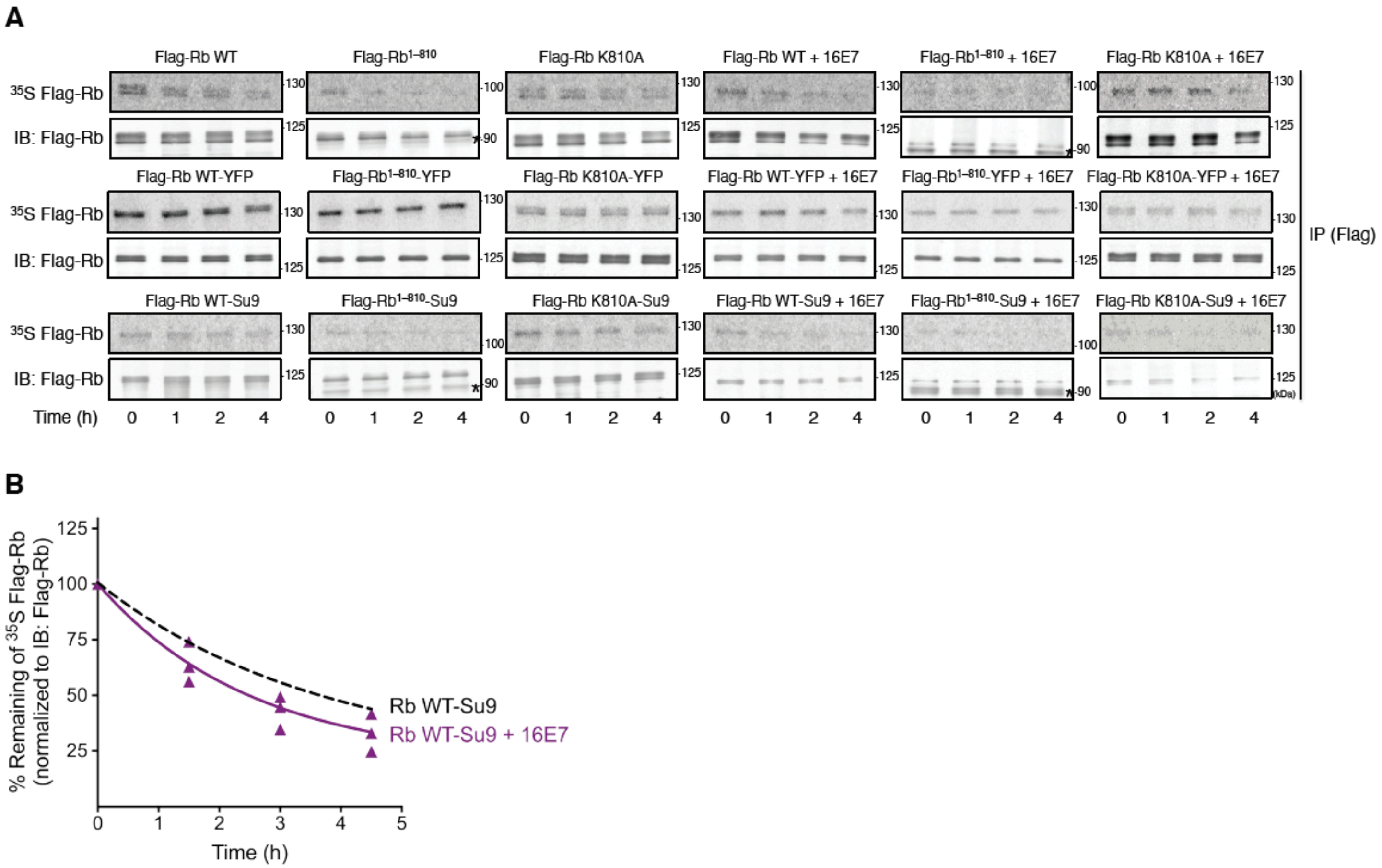

Supplementary Fig. 1. Metabolic pulse-chase of Rb mutants in HEK293T cells.
(A) Representative gel images for quantifications shown in Fig. 2 and Supplementary Fig. 1B. The asterisks denote non-specific bands. (B) Metabolic pulse-chase of Rb in cells as in Fig. 2. Effect of 16E7 expression on the degradation rate of Rb WT-Su9 was evaluated. Degradation of Rb-Su9 was replotted with broken line to help with comparison. The graph plots the relative band intensities from three repeat experiments. Source data are provided as a Source Data file.

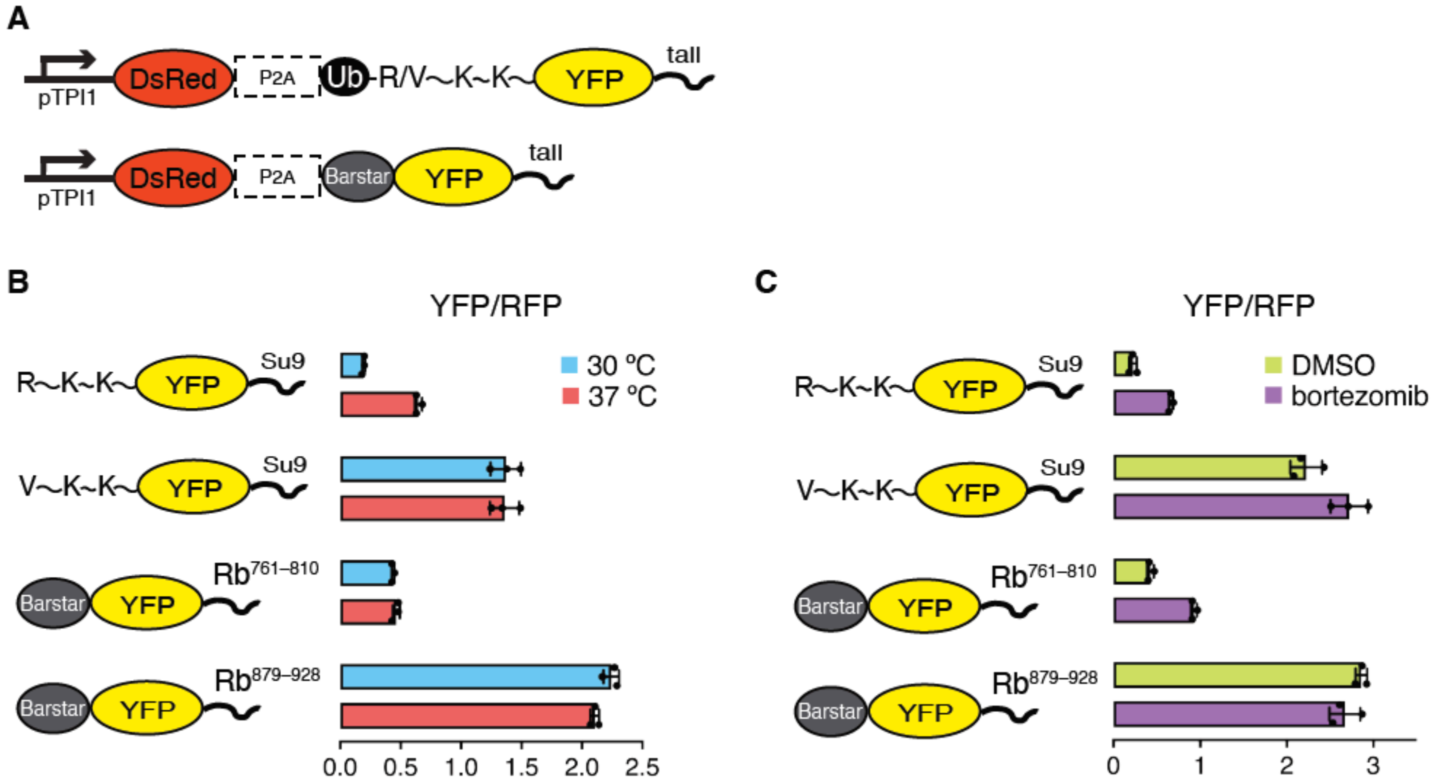

Supplementary Fig. 2. Initiation at the unmasked Rb tail is effective regardless of ubiquitination. (A) Schematic representation of fluorescence-based degradation assay in *S. cerevisiae* as in Fig. 3A. The substrate protein YFP-tail was fused to the C-terminal end of an N-end degron or the *B. amyloliquefaciens* barstar protein. (B) Cell fluorescence profiles of *S. cerevisiae* cultures expressing proteasome substrates with various initiation regions monitored by flow cytometry. The indicated substrates were expressed in the E1 temperature-sensitive strain *uba1-204* at the permissive temperature (30 ºC, blue) or the restrictive temperature (37 ºC, red). The graph shows relative median cellular YFP fluorescence (median YFP/RFP values) ± SD from triplicate experiments. (C) *S. cerevisiae* strain BY4741 carrying *pdr5*Δ expressing indicated proteasome substrates were treated with DMSO or 50 µM bortezomib for 6 h. Cell fluorescence profiles were monitored by flow cytometry. The graph shows relative median cellular YFP fluorescence (median YFP/RFP values) ± SD from triplicate experiments. Data are representative of three independent experiments. Source data are provided as a Source Data file.

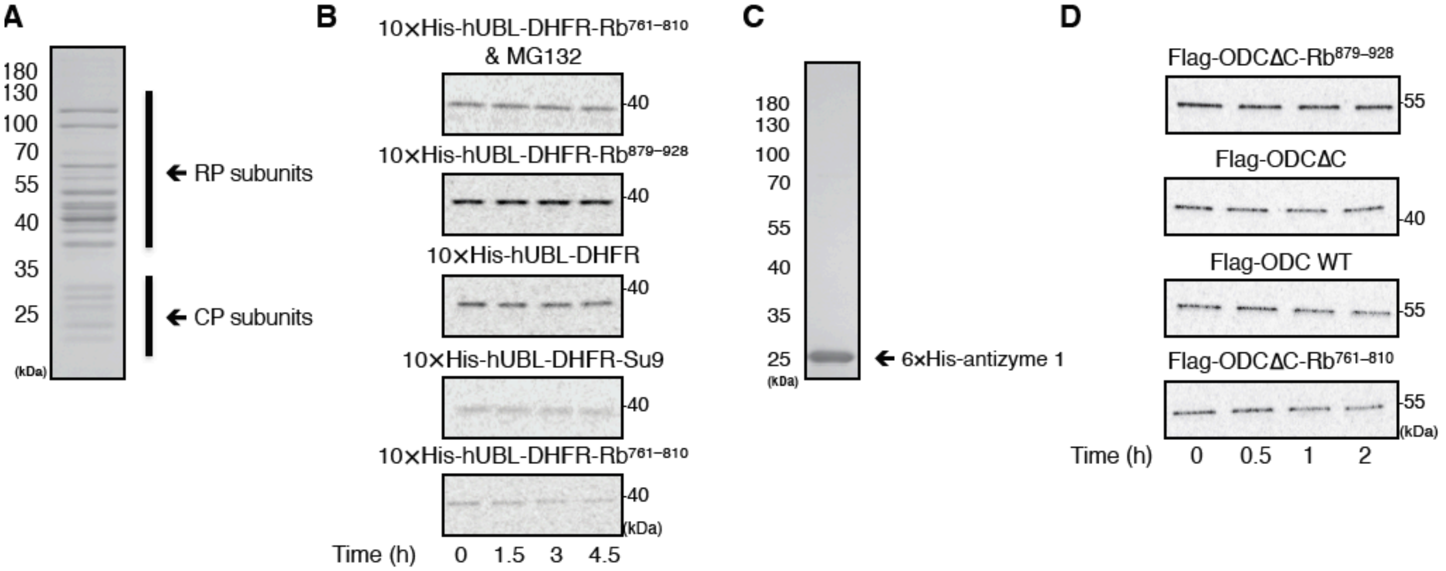

Supplementary Fig. 3. *In vitro* degradation assay using purified proteasome and antizyme.
(A) Purified mammalian proteasomes were analyzed by SDS-PAGE, followed by protein staining using InstantBlue. RP and CP denote regulatory particle and core particle, respectively. (B) Representative gel images corresponding to quantifications shown in Fig. 4B. (C) Purified 6×His-antizyme 1 were analyzed by SDS-PAGE, followed by protein staining using InstantBlue. (D) Representative gel images corresponding to quantifications shown in Fig. 4D. Data are representative of three independent experiments. Source data are provided as a Source Data file.

cleavage site

751 761 771 781 791 801
S I I V F Y N S V F M Q R L K T N I L Q Y A S T R P P T L S P I P H I P R S P Y K F P S S P L R I P G G N I Y I S P L K

811 821 831 841 851 861
S P Y K I S E G L P T P T K M T P R S R I L V S I G E S F G T S E K F Q K I N Q M V C N S D R V L K R S A E G S N P P K

871 881 891 901 911 921
P L K K L R F D I E G S D E A D G S K H L P G E S K F Q Q K L A E M T S T R T R M Q K Q K M N D S M D T S N K E E K

Supplementary Fig. 4. Amino acid sequence of the C-terminal region of Rb.
The residues from 751 to 928 of Rb are displayed. The calpain cleavage site (Lys 810), negatively charged residues (Asp [D] and Glu [E]), and $Rb^{879–888}$ as well as $Rb^{918–928}$ are highlighted in blue, red, and grey, respectively.

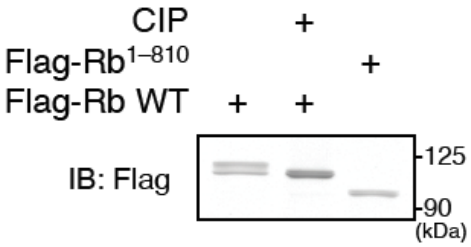

Supplementary Fig. 5. Rb WT is hyper-phosphorylated in cells.
HEK293T cells were transfected with Rb WT or Rb^{1-810} for 24 h. Cell lysates were treated with 20 U of calf intestinal alkaline phosphatase (CIP) at 37ºC for 1 h where indicated. Data is representative of three independent experiments. Source data are provided as a Source Data file.

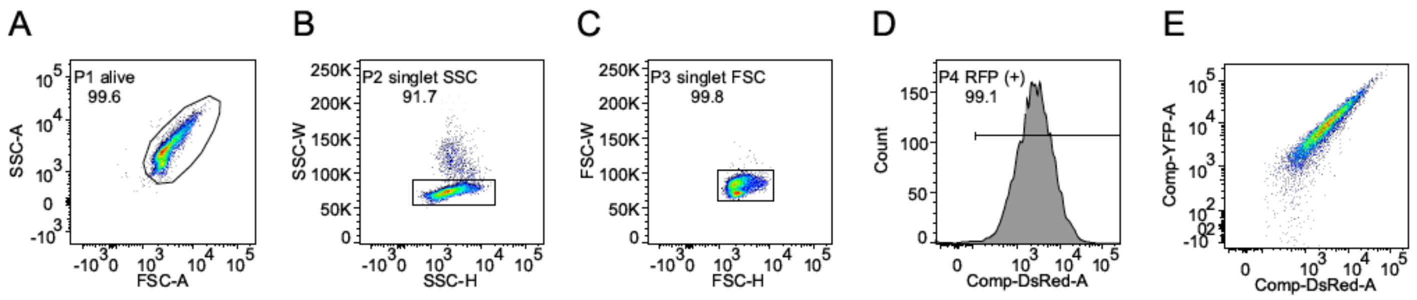

Supplementary Fig. 6. Gating strategy used for flow cytometry analysis.
(A–E) Representative scatter plots of flow cytometry analysis of yeast cells expressing RFP and YFP. Cells were gated along FSC-A/SSC-A axes (A) to eliminate dead cells and then gated along SSC-H/SSC-W (B) and FSC-H/FSC-W (C) to eliminate doublets, followed by gating of RFP-positive cells (D) to calculate the median YFP over RFP fluorescence ratios of the final population (E).

Tail	Amino acid sequences	Charge at pH 7.5	Theoretical pI	GRAVY (hydrophobicity)
SP2	RSPESMREEYRKEGSPESMREEYRKEGSPESM REEYRKEGSPESMREEYRKEGSPESMREEYRKE	-4.39	5.06	-2.363
eRR	GGGGAWLLPVSLVRRRTTLAPNTQTASPRALADS	2.61	12.00	-0.127
Su9	MASTRVLASRLASQMAASAKVARPAVRVAQVSKR TIQTGSPLQTRAYSS	7.61	12.31	-0.086
$Rb^{729-778}$	KDLPHAVQETFKRVLIKEEEYDSIIVFYNSVFMQRL KTNILQYASTRPPT	0.72	8.31	-0.416
$Rb^{761-810}$	MQRLKTNILQYASTRPPTLSPIPHIPRSPYKFPSSP LRIPGGNIYISPLK	6.72	11.10	-0.440
$Rb^{779-828}$	LSPIPHIPRSPYKFPSSPLRIPGGNIYISPLKSPYKI SEGLPTPTKMTPR	5.72	10.38	-0.488
$Rb^{829-878}$	SRILVSIGESFGTSEKFQKINQMVCNSDRVLKRSA EGSNPPKPLKKLRFD	3.78	10.01	-0.656
$Rb^{839-888}$	FGTSEKFQKINQMVCNSDRVLKRSAEGSNPPKPL KKLRFDIEGSDEADGS	-0.22	8.12	-0.982
$Rb^{849-898}$	NQMVCNSDRVLKRSAEGSNPPKPLKKLRFDIEGS DEADGSKHLPGESKFQ	-0.11	8.13	-1.134
$Rb^{859-908}$	LKRSAEGSNPPKPLKKLRFDIEGSDEADGSKHLP GESKFQQKLAEMTSTR	1.72	9.23	-1.206
$Rb^{869-918}$	PKPLKKLRFDIEGSDEADGSKHLPGESKFQQKLA EMTSTRTRMQKQKMND	2.72	9.54	-1.402
$Rb^{879-928}$	IEGSDEADGSKHLPGESKFQQKLAEMTSTRTRM QKQKMNDSMDTSNKEEK	-1.28	5.74	-1.596

Supplementary Table 1. Sequence profiles of initiation regions.
Amino acid sequences and bioinformatic data of initiation regions used in this study are shown.

Tail	Amino acid sequences	Charge at pH 7.5	Theoretical pI	GRAVY (hydrophobicity)
Rb^{1-52}	MPPKTPRKTAATAAAAAAEPPAPPPPPPPEEDPE QDSGPEDLPLVRLEFEET	-7.38	4.13	-0.967

Supplementary Table 2. Amino acid composition of the N-terminus of Rb.
The sequence profile of the N-terminus of Rb is shown as in Supplementary Table 1.

Name	Sequence	Description
16E7 BamHI F	GACTCCTATGGATCCATGCATGGAGATACACCTACATTGCATGAA	To amplify HPV 16E7
16E7 stop XhoI R	ATGCATCGCTCGAGTTATGGTTTCTGAGAACAGATGGGGCACAC	To amplify HPV 16E7
Rb BamHI F	ATAGGATCCATGCCGCCCAAAACCCCCCGAAAAAC	To amplify Rb
Rb stop XhoI R	TATCTCGAGTCATTTCTCTTCCTTGTTTGAGGTATCC	To amplify Rb
Rb aa810 stop XhoI R	AAACTCGAGTCACTTCAGGGGTGAAATATAGATGTTCCCTCCAG	To amplify cleaved Rb
Rb K810A F	CTATATTTCACCCCTGGCGAGTCCATATAAAATTTCAGAAG	To generate K810A mutation of Rb
Rb K810A R	GAAATTTTATATGGACTCGCCAGGGGTGAAATATAGATGTTC	To generate K810A mutation of Rb
Rb aa928 XhoI linker R	CTGAACCTGACTCGAGTTTCTCTTCCTTGTTTGAGGTATCC	To amplify Rb-linker to attach YFP
Rb aa810 XhoI linker R	CTGAACCTGACTCGAGCTTCAGGGGTGAAATATAGATGTTC	To amplify cleaved Rb-linker to attach YFP
Rb SacII Su9 R	ACGAGTGGAGGCCATCCGCGGTTTCTCTTCCTTGTTTGAGGTATCC	To attach Su9 tail to Rb
Rb aa810 SacII Su9 R	ACGAGTGGAGGCCATCCGCGGCTTCAGGGGTGAAATATAGATGTTC	To attach Su9 tail to cleaved Rb
Su9 F	ATGGCCTCCACTCGTGTCCTC	To amplify Su9 tail
Su9 stop NotI XhoI R	CTGACTCGAGCGGCCGCTTAGGAAGAGTAGGCGCGGG	To amplify Su9 tail
TPI1 SacI F	TACATAAACTAAAAGAGCTCATTAAAGAGGAG	To amplify insert in CEN plasmid
tail NotI tAHD R	TTAGAAGTTCTAGAGCGGCCGC	To amplify insert in CEN plasmid
Rb aa729 SacII F	ATACCGCGGAAGGATCTTCCTCATGCTGTTCAGG	To amplify tail derived from Rb
Rb aa778 stop XhoI R	ATACTCGAGTCAGGTAGGGGGCCTGGTGGAAGCATAC	To amplify tail derived from Rb
Rb aa761 SacII F	ATACCGCGGATGCAGAGACTGAAAACAAATATTTTG	To amplify tail derived from Rb
Rb aa779 SacII F	ATACCGCGGTTGTCACCAATACCTCACATTCCTCG	To amplify tail derived from Rb
Rb aa828 stop XhoI R	ATACTCGAGTCATCTTGGAGTCATTTTTGTTGGTG	To amplify tail derived from Rb
Rb aa829 SacII F	ATACCGCGGTCAAGAATCTTAGTATCAATTGGTG	To amplify tail derived from Rb
Rb aa878 stop XhoI R	ATACTCGAGTCAATCAAAGCGTAGTTTTTTCAGTGG	To amplify tail derived from Rb
Rb aa839 SacII F	ATACCGCGGTTCGGGACTTCTGAGAAGTTCCAG	To amplify tail derived from Rb
Rb aa888 stop XhoI R	ATACTCGAGTCAACTTCCATCTGCTTCATCTGATCC	To amplify tail derived from Rb
Rb aa849 SacII F	ATACCGCGGAATCAGATGGTATGTAACAGCGAC	To amplify tail derived from Rb
Rb aa898 stop XhoI R	ATACTCGAGTCACTGAAATTTGGACTCTCCTGGGAG	To amplify tail derived from Rb
Rb aa859 SacII F	ATACCGCGGCTCAAAAGAAGTGCTGAAGGAAGC	To amplify tail derived from Rb
Rb aa908 stop XhoI R	ATACTCGAGTCATCGAGTAGAAGTCATTTCTGCCAG	To amplify tail derived from Rb
Rb aa869 SacII F	ATACCGCGGCCTAAACCACTGAAAAAACTACGC	To amplify tail derived from Rb
Rb aa918 stop XhoI R	ATACTCGAGTCAATCATTCATTTTCTGCTTTTGCATTC	To amplify tail derived from Rb
Rb aa879 SacII F	ATACCGCGGATTGAAGGATCAGATGAAGCAGATG	To amplify tail derived from Rb
DsRed P2A R	TCCAGCCTGCTTCAGCAGGCTGAAGTTAGTAGCTCCGCTTCCCTGG AACAGGTGGTGGCG	To amplify DsRed-P2A
UBL P2A F	CTGCTGAAGCAGGCTGGAGACGTGGAGGAGAACCCTGGACCTGTT TCCCTGACGTTCAAA	To amplify P2A-UBL
P2A R	AGGTCCAGGGTTCTCCTCCAC	To amplify P2A
P2A Ub R	CACGAAGATCTGCATAGGTCCAGGGTTCTCCTCC	To amplify P2A-Ub
Ub F	ATGCAGATCTTCGTGAAGACTTTG	To amplify Ub
P2A Barstar F	GTGGAGGAGAACCCTGGACCTAAAAAAGCAGTCATTAACGGGGAAC AAATCAGAAG	To amplify P2A-Barstar
linker R	GGATCCACCGCCCGAAC	To amplify UBL-linker
linker F	GTCGACGGTGGTTCGGGC	To amplify linker-YFP
sYFP SacII R	GATAACCGCGGCTTGTACAGCTCGTCCATGC	To amplify YFP
10xHis-DHFR F	ATATACATATGCGGGGTTCTCATCATCATCATCATCATCATCATCATCA TGGTATTAG	To amplify 10xHis-DHFR
DHFR SacII R	GATAACCGCGGGCGGCGTTCCAGAATTTC	To attach tail to DHFR
DHFR stop SacII R	GATAACCGCGGTTAGCGGCGTTCCAGAATTTC	To amplify 10xHis-DHFR without tail
ODC BamHI F	ATAGGATCCATGAACAACTTTGGTAATGAAGAGTTTG	To amplify ODC
ODC stop XhoI R	ATACTCGAGCTACACATTAATACTAGCCGAAGCACAG	To amplify ODC
ODCΔC stop XhoI R	ATACTCGAGCTAGTCGGGGTTCTGGAATTGCTGCATG	To amplify ODC without tail
ODCΔC SacII R	ATACCGCGGGTCGGGGTTCTGGAATTGCTGCATG	To replace the tail of ODC
AZ1 EcoRI F	ATAGAATTCATGGTGAAATCCTCCCTGCAGCGGATCC	To amplify antizyme-1
AZ1 stop XhoI R	ATACTCGAGCTACTCCTCCTCCTCTCCCGAAGACTC	To amplify antizyme-1

Supplementary Table 3. Primers used in the study.
Primer sequences are given from the 5’-end to the 3’-end. “F” indicates the forward primer and “R” indicates the reverse primer.
