# Supplementary material for: A masked initiation region in retinoblastoma protein regulates its proteasomal degradation
Source: Nat Commun. 2020 Apr 24;11:2019. doi: 10.1038/s41467-020-16003-3 (PMC7181824; doi:10.1038/s41467-020-16003-3)
Supplement: Supplementary file 2 — Reporting Summary [file 41467_2020_16003_MOESM2_ESM.pdf]

nature research

Corresponding author(s): Andreas Matouschek

Last updated by author(s): Mar 26, 2020

Reporting Summary

Nature Research wishes to improve the reproducibility of the work that we publish. This form provides structure for consistency and transparency in reporting. For further information on Nature Research policies, see Authors & Referees and the Editorial Policy Checklist .

Statistics

For all statistical analyses, confirm that the following items are present in the figure legend, table legend, main text, or Methods section.

n/a	Confirmed	
☐	☒	The exact sample size (*n*) for each experimental group/condition, given as a discrete number and unit of measurement
☐	☒	A statement on whether measurements were taken from distinct samples or whether the same sample was measured repeatedly
☐	☒	The statistical test(s) used AND whether they are one- or two-sided<br>*Only common tests should be described solely by name; describe more complex techniques in the Methods section.*
☒	☐	A description of all covariates tested
☒	☐	A description of any assumptions or corrections, such as tests of normality and adjustment for multiple comparisons
☒	☐	A full description of the statistical parameters including central tendency (e.g. means) or other basic estimates (e.g. regression coefficient) AND variation (e.g. standard deviation) or associated estimates of uncertainty (e.g. confidence intervals)
☐	☒	For null hypothesis testing, the test statistic (e.g. *F*, *t*, *r*) with confidence intervals, effect sizes, degrees of freedom and *P* value noted<br>*Give P values as exact values whenever suitable.*
☒	☐	For Bayesian analysis, information on the choice of priors and Markov chain Monte Carlo settings
☒	☐	For hierarchical and complex designs, identification of the appropriate level for tests and full reporting of outcomes
☒	☐	Estimates of effect sizes (e.g. Cohen's *d*, Pearson's *r*), indicating how they were calculated

Our web collection on statistics for biologists contains articles on many of the points above.

Software and code

Policy information about availability of computer code

Data collection	Data were collected on an Odyssey (for western blotting, LI-COR), a Typhoon FLA 9500 (for autoradiography, GE Healthcare) and a LSRII Fortessa (for flow cytometry, BD Biosciences).
Data analysis	We used an ImageJ (for image quantification, NIH, ver 1.51), a FlowJo (for flow cytometry, BD Biosciences, ver 10.2) and a Snapgene (for sequence analysis, GSL Biotech, ver 4.2).

For manuscripts utilizing custom algorithms or software that are central to the research but not yet described in published literature, software must be made available to editors/reviewers. We strongly encourage code deposition in a community repository (e.g. GitHub). See the Nature Research guidelines for submitting code & software for further information.

Data

Policy information about availability of data

All manuscripts must include a data availability statement. This statement should provide the following information, where applicable:

- Accession codes, unique identifiers, or web links for publicly available datasets
- A list of figures that have associated raw data
- A description of any restrictions on data availability

The authors declare that the data supporting the findings of this study are available within the paper. The source data underlying Figs. 1C, 1D, 2B, 2C, 2D, 2E, 3B, 4B, 4D and Supplementary Figs. 1A, 1B, 2B, 2C, 3B, 3D, 5 are provided as a Source Data file. Other data that support the findings of this study are available from the corresponding author upon reasonable request.

Field-specific reporting

Please select the one below that is the best fit for your research. If you are not sure, read the appropriate sections before making your selection.

- [x] Life sciences
- [ ] Behavioural & social sciences
- [ ] Ecological, evolutionary & environmental sciences

For a reference copy of the document with all sections, see nature.com/documents/nr-reporting-summary-flat.pdf

Life sciences study design

All studies must disclose on these points even when the disclosure is negative.

Sample size	Sample sizes were chosen based on similar published studies elsewhere. All analyzes were performed at least three independent times and each repeat yielded highly similar results.
Data exclusions	Experiments were excluded from analysis when either the positive or negative control failed.
Replication	All the results were reproduced at least three times for each experiment.
Randomization	Randomization was not required because we performed all the experiments using specified cell lines.
Blinding	Blinding was not performed because there is no way to introduce bias into the experiments in this study.

Reporting for specific materials, systems and methods

We require information from authors about some types of materials, experimental systems and methods used in many studies. Here, indicate whether each material, system or method listed is relevant to your study. If you are not sure if a list item applies to your research, read the appropriate section before selecting a response.

Materials & experimental systems

n/a	Involved in the study	
[ ]	[x]	Antibodies
[ ]	[x]	Eukaryotic cell lines
[x]	[ ]	Palaeontology
[x]	[ ]	Animals and other organisms
[x]	[ ]	Human research participants
[x]	[ ]	Clinical data

Methods

n/a	Involved in the study	
[x]	[ ]	ChIP-seq
[ ]	[x]	Flow cytometry
[x]	[ ]	MRI-based neuroimaging

Antibodies

Antibodies used	Anti-Flag (1:1,000; Sigma #F1804), anti-actin (1:1,000; Sigma #A2066), and anti-16E7 (1:1,000; Santa Cruz #sc-51951).
Validation	Anti-Flag; the manufacturer validated that this antibody detects only the band(s) of Flag-tagged protein on a Western blot from an E. coli, plant or mammalian crude cell lysate.<br>Anti-actin; the manufacturer validated that this antibody recognizes the 42 kDa actin band using immunoblotting with human or animal tissue extracts.<br>Anti-16E7; the manufacturer validated that this antibody recognizes HPV16 E7 protein expressed in the HPV16-positive Caski cell line on a Western blot.

Eukaryotic cell lines

Policy information about cell lines

Cell line source(s)	HEK293T (ATCC) and Rpn11-Flag MEFs (Shigeo Murata, the University of Tokyo)
Authentication	Not authenticated.
Mycoplasma contamination	All cell lines tested negative for micoplasma contamination.
Commonly misidentified lines (See ICLAC register)	No commonly misidentified cell lines were used in the study.

Flow Cytometry

Plots

Confirm that:

- [x] The axis labels state the marker and fluorochrome used (e.g. CD4-FITC).
- [x] The axis scales are clearly visible. Include numbers along axes only for bottom left plot of group (a 'group' is an analysis of identical markers).
- [x] All plots are contour plots with outliers or pseudocolor plots.
- [x] A numerical value for number of cells or percentage (with statistics) is provided.

Methodology

Sample preparation	Yeast cells were cultured to early log phase before harvesting for analysis.
Instrument	LSRII Fortessa (BD Biosciences)
Software	FlowJo v.10.2 (FlowJo LLC)
Cell population abundance	The abundance of the desired cell population in post-gating was generally > 95% of the total cells.
Gating strategy	Living singlet yeast cells were determined by their sizes (FSC/SSC) and RFP-positive cells were gated for measurement of YFP over RFP fluorescence ratio of the final population.

- [x] Tick this box to confirm that a figure exemplifying the gating strategy is provided in the Supplementary Information.
